# Soft Tissue Necrosis in Head and Neck Cancer Patients After Transoral Robotic Surgery or Wide Excision With Primary Closure Followed by Radiation Therapy: Erratum

**DOI:** 10.1097/01.md.0000484087.58594.49

**Published:** 2016-05-20

In the article “Soft Tissue Necrosis in Head and Neck Cancer Patients After Transoral Robotic Surgery or Wide Excision With Primary Closure Followed by Radiation Therapy”,^1^ which appeared in Volume 95, Issue 9 of *Medicine*, Dr. Yun Hee Lee's name was originally spelled incorrectly. The article has since been corrected online.
